# Midwives’ descriptions of avoidable causes of negative perinatal outcomes

**DOI:** 10.4102/hsag.v28i0.2185

**Published:** 2023-07-05

**Authors:** Kagiso P. Tukisi

**Affiliations:** 1 Department of Nursing Science, School of Health Care Sciences, Sefako Makgatho Health Sciences University, Pretoria, South Africa

**Keywords:** midwives, labour, maternal mortality, medical personnel-related avoidable causes, neonatal mortality, perinatal outcomes

## Abstract

**Background:**

The Perinatal Problem Identification Programme (PPIP) is used to rule out the avoidable and nonavoidable causes of negative maternal and perinatal outcomes through file audits. Perinatal Problem Identification Programme serves as a tool for midwives and obstetricians to pinpoint missed opportunities that could prevent avoidable causes of negative perinatal outcomes.

**Aim:**

The study aimed to describe and explore the avoidable causes of negative perinatal outcomes in Bojanala District through the lens of the midwife.

**Setting:**

This study was conducted in the two selected facilities in the Bojanala District in the North West Province of South Africa.

**Methods:**

The study derived from a larger study that focused on midwives’ experiences of obstetric triage in the Bojanala District. A qualitative, exploratory and descriptive research design was used with purposive sample of nine midwives. Participants had over 5 years of clinical midwifery experience and were employed in the Bojanala District. Semi-structured interviews were utilised with data analysed using Colaizzi’s descriptive method of data analysis.

**Results:**

Three major themes with eight subthemes emerged. Midwives noted space constraints, medicine and medical supply constraints, and constraints in availability of medical equipment. Access to identified constraints would enable prompt and appropriate management.

**Conclusion:**

The study highlighted the experience of midwives in accessing needed space, medicines, medical supplies and equipment, potentially impacting negative perinatal outcomes.

**Contribution:**

This study provides insight into administratively related avoidable causes of negative perinatal outcomes through the lens of frontline maternity care providers – midwives. Findings should be of particular utility to health service managers working to reduce maternal mortality and morbidity.

## Introduction

The Perinatal Problem Identification Programme (PPIP) is a tool that makes perinatal and maternal death audits easier to conduct. Introduced in 1999, the tool allows for monthly analysis of avoidable factors as it relates to deaths and examines the number of deliveries, stillbirths, early neonatal deaths and causes of such deaths (Department of Health [DOH] n.d.). The perinatal outcomes of pregnancy and labour in the PPIP context are classified as negative or positive (DOH 2021). The PPIP analyses also provide insights into avoidable and nonavoidable causes of the negative perinatal outcomes (Moodley, Fawcus & Pattinson 2020). Perinatal Problem Identification Programme identifies gaps in maternal and perinatal care and accurately records the causes of maternal and neonatal deaths so that guidelines can be developed to address such gaps (DOH n.d.). The PPIP serves as a quality improvement tool in midwifery care.

Use of the PPIP serves as one strategy to help reach two important sustainable development goals (SDGs) set by the World Health Organization (WHO 2022). These two target goals include ‘reducing the global maternal mortality ratio to less than 70 per 100 000 live births’ and to ‘end preventable deaths of newborns and children under five years of age and reduce neonatal mortality to at least 12 per 1000 births’. Despite efforts since 2015 to meet WHO SDGs, maternal and neonatal mortalities in South Africa continue to rise and are mainly due to avoidable causes (Statistics South Africa 2022).

Avoidable factors within the PPIP context refer to incidents related to actions taken by the mother, healthcare personnel or the health system, which may have altered the perinatal outcomes. Avoidable factors are classified as *probable* or *possible* contributors to the cause of death (Moodley et al. 2020). Negative perinatal outcomes are mainly due to inappropriate responses to existing and potential problems (Vallely et al. 2021). Midwives are frontline in care of the labouring woman in public facilities in South Africa and are tasked with the responsibility of identifying both existing and potential problems. Accurate identification of such problems serves as the basis for midwifery care plans with the potential to limit missed opportunities that could give rise to deleterious outcomes.

The overall mortality rate in South Africa is on the rise, and this has been associated with 5Hs (haemorrhage, hypertension, HIV, health personnel and health system strengthening) (DOH 2016). Ongoing PPIP audits for both maternal and neonatal deaths in South Africa have demonstrated that the 5Hs are avoidable factors and are largely related to facility level issues which account for the majority of perinatal deaths (Moodley, Fawcus & Pattinson 2020). To date, hypertensive-related disorders in pregnancy are the second leading cause of maternal mortality (DOH 2016). However, PPIP audits reveal that the majority of the hypertensive-related deaths could have been avoided if hypertension had been diagnosed early in pregnancy and/or labour with prompt management instituted (Lavin et al. 2018). Obstetric haemorrhage remains the leading cause of the maternal mortality (Heitkamp et al. 2020) with PPIP audits demonstrating that it is largely avoidable when anticipated (DOH 2021).

Perinatal Problem Identification Programme audits reveal that the majority of recorded perinatal mortalities are the result of poor foetal surveillance (Thapa & Sah 2017). In some instances where there was perinatal death, there was no evidence of foetal heart rate monitoring on admission (DOH 2021). This finding was associated with failure to perform an admission cardiotocograph tracing and a lack of baseline data (Bera et al. 2022). In about 23% of the recorded perinatal deaths, according to the PPIP audit, the foetal heart rate was monitored but there was no interpretation, which led to an inappropriate response by the midwives (Marombwa et al. 2019). Audits raised an even greater concern as it suggested that there is a contravention of the prescribed foetal monitoring guidelines during labour (DOH 2016). This may have resulted in a decline in the quality of foetal monitoring and care during labour as evidenced by the alarming perinatal mortality rates.

The detrimental effects of missed opportunities often lead to midwives being charged with negligence and potential litigation against them (Du Plessis, Grobler & Seekoe 2020). Litigations are often well-documented as the result of missed opportunities for the midwife to accurately diagnose, stabilise or treat, and refer (Du Plessis et al. 2020). The reasons underlying the avoidable causes of negative perinatal outcomes, unfortunately, are not well explained in the literature. Midwives rendering care can provide valuable insight into the occurrence of negative perinatal outcomes where causes are avoidable.

This study aimed to describe the avoidable causes of negative perinatal outcomes through the lens of midwives. Study findings provide insight into administratively related avoidable causes of negative perinatal outcomes as described by midwives. These findings could be of assistance to health services managers as they plan and allocate resources important in the reduction of perinatal mortality.

## Research purpose

This study examined the avoidable causes of negative perinatal outcomes in selected facilities in Bojanala District, North West Province, through the lens of the midwife.

## Research design

A qualitative, exploratory, descriptive research design was employed to uncover midwives’ experiences of avoidable causes of negative perinatal outcomes.

### Research setting and context

A setting in the research context refers to the exact location where the study and data collection take place (Polit & Beck 2019). The study was conducted in the Rustenburg sub-district, one of the five sub-districts located within the Bojanala district of the North West Province of South Africa. Rustenburg health sub-district consists of 21 primary healthcare clinics, 3 midwife-led obstetric units (MOUs), 2 district hospitals and 1 tertiary hospital. The tertiary hospital is the only main referral hospital for the 21 clinics, 3 MOUs and the 2 district hospitals in the Bojanala District.

The facilities that were under study were the tertiary hospital and one selected MOU at the time of the study. The selected MOU had 11 midwives and the hospital had 27 midwives. The selected facilities offer 24-h midwifery services to the population residing in Bojanala District. During data collection, the selected tertiary hospital averaged 750 births monthly; the MOU handled approximately 350 births monthly. To sum up the population among the two selected facilities, there were a total of 38 midwives and approximately 1100 births monthly.

### Sampling process

Prospective participants were accessed through their immediate managers who served as gatekeepers. Research invitation letters were presented to the gatekeepers and the operational managers identified the prospective participants. Participants indicated that interviews at work would be an inconvenience as it interrupted care to women in labour. Consequently, participants arranged an appointment with the researcher during their off time and in their own homes. The researcher acknowledged the decision as it was convenient for the participants. A purposive sampling was used to select participants who met the following inclusion criteria:

- Five years or more of full-time clinical midwifery experience.
- Full-time employment.
- Registered with the South African Nursing Council (SANC) as a midwife and (or) with additional midwifery qualification.

Initially, 12 midwives who met the inclusion criteria were recruited into the study; and 9 midwives were included. Those who did not participate were excluded as they did not meet inclusion criteria. Exclusion criteria included:

- Less than 5 years clinical midwifery experience.
- Employment in other units within the selected facilities.
- Qualified midwives not yet registered by the SANC.

### Data collection

Data collection took place between February and May 2019 following receipt of ethical clearance. The first two participants formed part of the pilot study where the aim was to check the adequacy and simplicity of the research question and the need to refine the question. The research question proved to be understandable to the participants and data received from the pilot study were consistent with data obtained from subsequent interviews. Data from the pilot study were included in the main study. The participants from the pilot study formed part of the main sample.

Participants engaged in a semi-structured audio-recorded interview with the study researcher. Interviews with each participant lasted between 35 and 60 min. The central question asked of all participants was, ‘In your view, what are the avoidable causes of negative perinatal outcomes in your facility?’

Probing questions were used to encourage greater detail and depth of midwifery responses. The following questions were used as probes:

- You are saying … What was the situation?
- Give me more detail about …, please.
- When you say … What more do want to see being done about the avoidable causes?
- Can you think of another example of this?
- Looking back, what would you do differently from now about the avoidable causes, if anything?

Interviews were transcribed verbatim by the researcher. Transcriptions were completed on the day of the interview to avoid overlooking other important information.

### Data analysis

The researcher employed Collaizi’s seven-step descriptive phenomenological method of data analysis (Polit & Beck 2017). This method provided the framework for describing the avoidable causes of negative perinatal outcomes as viewed by midwives. The data analysis process proceeded as follows:

#### Step 1: Read the transcriptions to acquire a feeling for them

Following verbatim transcription and translation, the researcher read the transcriptions multiple times to gain a real feel and understanding of midwives’ descriptions of avoidable causes of negative perinatal outcomes in their respective facilities.

#### Step 2: Extract significant statements

The researcher reviewed each transcription and extracted significant statements that directly related to the avoidable causes of negative perinatal outcomes.

#### Step 3: Formulate meanings of each significant statement

The researcher referred back to the transcriptions and formulated meanings from the significant statements relating to avoidable causes of negative perinatal outcomes to discover the hidden, real and original meanings.

#### Step 4: Organise the formulated meanings into clusters of themes

The formulated meanings were then organised into themes by an independent coder who was an expert in qualitative data analysis.

#### Step 5: Integrate results into exhaustive meanings

Results of midwives’ descriptions of avoidable causes of negative perinatal outcomes were integrated into exhaustive meanings, which provided a complete description.

#### Step 6: Formulate an exhaustive description

The cluster of themes that emerged from the formulated meanings was integrated to form an exhaustive description of the avoidable causes of the negative perinatal outcomes.

#### Step 7: Validate

Participants were granted an opportunity to read the exhaustive descriptions as a means of validating participant descriptions of avoidable causes of negative perinatal outcomes. The researcher also extended an invitation to participants to make additions they deemed as important for inclusion in the descriptions.

Inductive reasoning was employed as the researcher reflected on participant experiences (Polit & Beck 2017). To eliminate bias, the researcher disclosed that he is an experienced advanced accoucheur and a neonatal nurse. The researcher bracketed his own thoughts and ideas and reported data as expressed by the participants based on their own experiences using direct quotations. An independent coder was also employed to eliminate bias and to formulate final themes. Final themes were also reviewed by the investigator’s research supervisors who were experts in both midwifery care and qualitative research methodology.

### Trustworthiness

The researcher employed Lincoln and Guba’s (1986) general criteria to ensure trustworthiness (Polit & Beck 2019). These principles included credibility, transferability, dependability, confirmability and authenticity. As a measure to maintain the credibility of the study, the researcher adhered to the approved research protocol and only participants who met the inclusion criteria were enrolled. To remain consistent in the collection of data, the researcher employed a semi-structured interview with one central question followed by probing questions, which ensured prolonged engagement. Prolonged engagement with participants aided in persistent observation, member checking and peer debriefing. To increase dependability of the study, participants were interviewed until data saturation was achieved. Data saturation was reached when themes and categories of collected data become repetitive and redundant with no new information emerging (Polit & Beck 2017:744). The researcher conducted preliminary data analyses with an independent coder and then employed to ensure the quality of the data and the analysis. The independent coder and the researcher met to compare and discuss data to reach consensus about the themes. Amidst data collection, the researcher also kept an audit trail and collected field notes, which were included in the data analysis and the final report providing further evidence of authenticity (Creswell & Poth (2018).

To ensure transferability and generalisability of findings, the researcher provided a dense description of the demographic information related to the study. The researcher used direct quotations from participants, ensuring a rich description of midwives’ perceptions. To ensure confirmability, an audit trail of all documentation regarding the research study, that is, all audio-taped material, written notes as well as the verbatim transcribed audio notes, were kept safely under lock and key and accessible only to the researcher. Finally, to ensure authenticity of the study, the researcher captured in text the real feelings, mood, experience and language as expressed by the participants which would enhance understanding of the feel of the context by participants (Polit & Beck 2019:560).

### Ethical considerations

Prior to conducting the study, a proposal was submitted and approved by the University of Johannesburg’s Research Ethics and the Higher Degree Committee. The study was registered in the National Health Research Database (NHRD) which approved the application to be forwarded to the North West Department of Health (NWDOH). Once the NWDOH approved the study, the researcher made applications for access to relevant facilities in the Bojanala district. The study included human subjects as participants and therefore the researcher adhered to the Declaration of Helsinki (Sawicka-Gutaj et al. 2022). The researcher provided the prospective participants with detailed research information to gain their informed consent. To protect their confidentiality and ensure anonymity, the researcher generated codes to be used during the discussion of data. The study was deemed to pose minimal risk to participants, though emotional distress as potentially painful experiences were recounted was a possibility. Ethical clearance was received prior to study recruitment and enrollment (UJrec 241112-035; HDC-01-117-2018).

## Results

Data were collected from a sample of nine midwives with clinical midwifery experience that ranged from 5 to 18 years. All were female between 27 and 52 years of age, black and held a bachelor’s or diploma in midwifery (see Table 1).

**TABLE 1:** Summary of description of the participants.

Code	Age	Gender	Ethnicity	Qualifications	Experience (years)
P1	27	Female	Black people	Bachelor of Nursing and Midwifery	5
P2	40	Female	Black people	Diploma in Nursing and Midwifery	16
P3	42	Female	Black people	Diploma in Nursing and Midwifery	8
P4	52	Female	Black people	Diploma in Nursing and Midwifery Diploma in Advanced Midwifery	18
P5	27	Female	Black people	Diploma in Nursing and Midwifery	5
P6	40	Female	Black people	Diploma in Nursing and Midwifery Diploma in Advanced Midwifery	12
P7	40	Female	Black people	Bachelor of Nursing and Midwifery	16
P8	26	Female	Black people	Bachelor of Nursing and Midwifery	5
P9	36	Female	Black people	Diploma in Nursing and Midwifery	7

*Source:* Tukisi, K.P., 2019, ‘The experience of midwives with regard to the use of obstetric triage by midwives in Bojanala District’, Master’s dissertation, University of Johannesburg, Johannesburg, viewed 03 March 2022, from https://hdl.handle.net/10210/412370.

Three primary themes and related categories were identified upon data analysis. These themes reflected participant descriptions of the space and material constraints which contributed to avoidable causes of negative perinatal outcomes (see Table 2).

**TABLE 2:** Summary of description of themes and subthemes.

Themes	Subcategories
1. Space constraints in admission, delivery rooms and high-care areas	1.1. Space constraints in admission rooms 1.2. Space constraints in the delivery rooms 1.3. Space constraints in high-care areas
2. Medicines and medical supplies constraints	2.1. Constraints in medicine supply 2.2. Constraints in medical supplies
3. Constraints in supply of the medical equipment for maternal, foetal and neonatal care	3.1. Constraints in medical equipment meant for maternal care 3.2. Constraints in medical equipment meant for foetal monitoring and care 3.3. Constraints in medical equipment meant for neonatal care

*Source:* Tukisi, K.P., 2019, ‘The experience of midwives with regard to the use of obstetric triage by midwives in Bojanala District’, Master’s dissertation, University of Johannesburg, Johannesburg, viewed 03 March 2022, from https://hdl.handle.net/10210/412370.

The central theme that emerged was that midwives were frustrated by the perceived serious constraint in space, supplies and equipment that were necessary for midwifery care. Participants associated these constraints as an avoidable reason where there were negative perinatal outcomes for mothers and babies, according to PPIP.

### Theme 1: Space constraints in admission, delivery rooms and high-care areas

Midwives were aware of the high influx of patients accessing their facilities for midwifery care and inadequate infrastructure.

#### Sub-category 1.1: Space constraints in admission rooms

Midwives noted that there were space constraints in the admission rooms meant for triage of patients:

‘Our admission room is a four-bed room, sometimes it will be full.’ (P2, Female, Black person, Diploma in nursing & midwifery)

‘We are only working with four beds and patients must line up on benches, [*with*] some of them sitting on the floor or outside because there is no room …’ (P7, Female, Black person, Bachelor of nursing & midwifery)

Space constraints in admission rooms were associated with a delayed response to patients by midwives:

‘We fail to manage them accordingly on time because of the lack of space.’ (P6, Female, Black person, Diploma in nursing & midwifery, Diploma in advanced midwifery)

The delayed response to women queuing for assessments in admission rooms made triage and prioritisation of women very difficult to accomplish:

‘You find that you try to prioritize patients but then you find that maybe there is *Er! Er!* Precipitated labour in admission while waiting.’ (P5, Female, Black person, Diploma in nursing & midwifery)

Space constraints in admission rooms were exacerbated by the fact that some patients needed more time than others:

‘Sometimes you’ve got a patient who is bleeding on top of the bed … or a patient with severe pre-eclampsia. You cannot take her out of the bed, you see. So you will only be working with two beds or sometimes with one bed, because one of the patients will be having a non-reassuring CTG or foetal distress so she will be on oxygen.’ (P7, Female, Black person, Bachelor of nursing & midwifery)

Midwives explained that a delayed response to women and delayed prioritisation of health needs were due to space constraints and caused an increase in waiting time. Such delay had the potential to exacerbate the patient condition:

‘Other patients are complicating while still waiting on the benches. We fail to manage them accordingly on time because of the lack of space.’ (P6, Female, Black person, Diploma in nursing & midwifery, Diploma in advanced midwifery)

#### Sub-category 1.2: Space constraints in the delivery rooms

Midwives explained that the delivery rooms were just not adequate for the volume of women they were seeing at the facility:

‘We only have a 4 bedded labour room in this facility. Mind you, we are receiving all the referrals from the clinics and MOU’s.’ (P6, Female, Black person, Diploma in nursing & midwifery, Diploma in advanced midwifery)

Participants associated space constraints with an inability to assess problems well in advance or to monitor the progress of labour:

‘There is just no space to monitor the woman during the active phase of labour.’ (P3, Female, Black person, Diploma in nursing & midwifery)

‘Sometimes you’ve got a patient who is bleeding on top of the bed, you cannot immediately transfer them … You know that her PV [*vaginal*] examination is due but there is no available bed for you to do it. You need to wait for another one to deliver so that you can have the bed.’ (P7, Female, Black person, Bachelor of nursing & midwifery)

Poor foetal heart rate monitoring was associated with compromise of patient safety:

‘Sometimes it is foetal distress, and you don’t even know because you don’t even know where to put the patient …’ (P7, Female, Black person, Bachelor of nursing & midwifery)

Midwives noted that women sometimes give birth unsupervised because they could not be monitored vigilantly:

‘Some of the women end up delivering on those benches while waiting for an available bed, and now we have to rush to save the newborn.’ (P9, Female, Black person, Diploma in nursing & midwifery)

Limited space in the labour room compromised not only safety but also the dignity and privacy of women:

‘There is no privacy in our labour rooms, because you will find that there are two women in one labour room, and then when you deliver this one and you close the curtain, but still that one can see what is happening.’ (P6, Female, Black person, Diploma in nursing & midwifery, Diploma in advanced midwifery)

#### Sub-category 1.3: Space constraints in high-care areas

Midwives recognised the level of care they were operating under and were aware of the complexity of the conditions of women they were treating:

‘The hospital we are working in is the only hospital in this area and we are receiving referred cases from the clinics and MOU. We are supposed to manage all the high-risk patients.’ (P6, Female, Black person, Diploma in nursing & midwifery, Diploma in advanced midwifery)

Participants noted that despite the hospital being the only referral facility equipped to manage women with complex obstetrical conditions, space constraints made the required increased vigilance difficult:

‘We have space for six patients in high care and you find that the sister who is working in high care now is seeing eight patients, who are critically ill.’ (P7, Female, Black person, Bachelor of nursing & midwifery)

‘We now need to open the side room and use it as high care for the two patients. The room is an ordinary room without the high care equipment needed.’ (P1, Female, Black person, Bachelor of nursing & midwifery)

### Theme 2: Medicines and medical supply constraints

Midwives seemed to be conversant with the pharmacological management of obstetric complications as well as necessary life-saving interventions. However, they were concerned about the constraints in medicines and medical supplies.

#### Sub-category 2.1: Constraints in medicines supply

Participants noted that some of the drug interventions that needed to be administered were missing or in short supply. Absence of life-saving drugs made management very difficult:

‘The patient has to get treatment immediately. We can’t wait for the patient to be admitted in the ward first because we need to stabilize the condition before we send them to the ward.’ (P6, Female, Black person, Diploma in nursing & midwifery, Diploma in advanced midwifery)

‘… with PPH, you will need to immediately start with the obstetric management, you need oxytocin to run per drip … but there wasn’t any oxytocin available.’ (P5, Female, Black person, Bachelor of nursing & midwifery)

‘Sometimes you are dealing with preterm labor, then you find that even tocolytics [*are unavailable*] … there is nothing. How are you going to do tocolysis [*for*] the patient now?’ (P4, Female, Black person, Diploma in nursing & midwifery, Diploma in advanced midwifery)

Constraints in medicine supply also interfered with the continuity of care women received:

‘Sometimes you have a patient who has already received the loading dosage of magnesium sulphate either at the MOU or clinic. You may find that there is no magnesium sulphate available in the tertiary hospital … but the referral centers have it.’ (P4, Female, Black person, Diploma in nursing & midwifery, Diploma in advanced midwifery)

‘So, the patient ends up fitting because the blood pressure is not controlled. The blood pressure is above 160/110. Then the patient will have to be delivered, irrespective of the gestational age.’ (P8, Female, Black person, Bachelor of nursing & midwifery)

#### Sub-category 2.2: Constraints in medical supply

In addition to the constraints in medicines and necessary drugs for necessary for midwifery care, participants explained that they found it difficult to carry out their tasks due to constraints in medical supplies:

‘There is nothing that hurts like, you know, what is it that you are supposed to be doing but you cannot because you don’t have the proper material.’ (P8, Female, Black person, Bachelor of nursing & midwifery)

Participants mentioned that they often lacked even the basic medical supplies such as oxygen masks, needed during resuscitation:

‘We lack almost everything because, for example, mere oxygen masks might not be there, which is very crucial for intrauterine resuscitation.’ (P4, Female, Black person, Diploma in nursing & midwifery, Diploma in advanced midwifery)

They associated constraint in medical supplies to maternal and neonatal deaths:

‘You end up having foetal distress that ends up being FSB’s [*Fresh stillborn*] … you can easily lose the mother if you are not fully equipped for resuscitation.’ (P8, Female, Black person, Bachelor of nursing & midwifery)

### Theme 3: Constraints in supply of the medical equipment for maternal, foetal and neonatal care

Midwives seemed to be conversant with the vigilant monitoring of both the mother and the foetus during various stages of care of the woman. However, they were concerned about the constraints in the supply of the reliable medical equipment to enable them to work.

#### Sub-category 3.1: Constraints in medical equipment for maternal care

Midwives were knowledgeable and conversant with responsibilities required during admission and care of the woman in labour:

‘When you admit the patient you must do everything for the patient, you must assess and plan for the patient.’ (P3, Female, Black person, Diploma in nursing & midwifery)

‘On admission when we triage a patient we are supposed to do all the vital signs which means we will have to check the BPs, the HGTS’s and the HB’s … those are the most important things that we are supposed to do.’ (P5, Female, Black person, Diploma in nursing & midwifery)

Midwives noted the equipment necessary equipment to care for women on admission, as well as during labour:

‘When you admit a patient, I think the resources must be up to standard! BP machines, CTG, rapid testing machines.’ (P7, Female, Black person, Bachelor of nursing & midwifery)

Midwives were troubled by the constraints in medical equipment used to assess the women’s condition:

‘We don’t have enough equipment to do a proper diagnosis and assessment of the patient.’ (P9, Female, Black person, Diploma in nursing & midwifery)

‘We’ve got only one BP machine that will be running in the whole ward.’ (P1, Female, Black person, Bachelor of nursing & midwifery)

Participants were concerned that constraints were the reason for increased waiting times which contributed to increased complications:

‘You will find that the machine is not working – especially the HB [*Haemoglobin*] machine, … you see that the patient is pale and you cannot really check the patient immediately. Now you have to wait for a formal HGT [*Haemoglucotest*] machine … the formal HB to be done and it takes time … meanwhile, the woman is complicating.’ (P5, Female, Black person, Diploma in nursing & midwifery)

They also explained that constraint in medical supplies was the reason why some important diagnostic information was not obtained:

‘You find that the haemoglucotest (HGT) monitor is not working, and you have to admit patient with gestational diabetes mellitus who really needs an HGT on admission. The HGT results will be missing in the patient’s file because you are unable to monitor.’ (P5, Female, Black person, Diploma in nursing & midwifery)

Midwives explained that due to the experienced constraints in medical equipment – equipment that was constantly in operational and was seldom serviced – there were feelings of mistrust:

‘Our machines are few and aren’t working – nonstop. Sometimes you find that they are not working properly, so sometimes you doubt if the BP or pulse you are seeing is really that of the patient.’ (P3, Female, Black person, Diploma in nursing & midwifery)

‘Sometimes the machine takes time to be serviced. At times you are not so sure if the readings that they are giving you are really what is happening with the patient.’ (P9, Female, Black person, Diploma in nursing & midwifery)

#### Sub-category 3.2: Constraints in medical equipment meant for foetal monitoring and care

Midwives recognised a need to do electronic foetal monitoring on all pregnant women being admitted and progressing in labour. They recognised that constraints in medical equipment for foetal monitoring made it impossible for vigilant monitoring:

‘You find that you have only 3 CTG’s [*Cardiotocograph machines*], with a 24 bedded maternity and labour [*unit*].’ (P4, Female, Black person, Diploma in nursing & midwifery, Diploma in advanced midwifery)

‘I don’t have resources and then sometimes the woman will be having foetal distress and you have to do a continuous CTG … I have 15 women waiting to have a CTG. How am I going to do it?’ (P4, Female, Black person, Diploma in nursing & midwifery, Diploma in advanced midwifery)

Participants noted that the cardiotocographs in use were not fully serviced and therefore often provided inaccurate readings. When coupled with a shortage of equipment, potential inaccuracy in data made diagnoses very difficult, increased patient waiting time and contributed to negative perinatal outcomes:

‘Sometimes the CTG, the one that has to give us the proper diagnosis … sometimes it loses contact, sometimes the toco is not working, you cannot tell if the patient is really in labour … you can only trace the foetal heart. You cannot really tell if the patient is really in labour.’ (P9, Female, Black person, Diploma in nursing & midwifery)

‘The CTG has to run at least thirty minutes for you to make a proper diagnosis on the fetus and the mother as well as to establish true labour. So, with a slow CTG it would mean you have to run it for almost fifty minutes to get a trace that you can interpret.’ (P9, Female, Black person, Diploma in nursing & midwifery)

‘… because of [*a*] shortage of equipment it makes it even more difficult for you (the midwife) and you end up having maternal deaths, you end up having foetal distress … that [*often*] ends up being FSB’s.’ (P8, Female, Black person, Bachelor of nursing & midwifery)

#### Sub-category 3.3: Constraints in medical equipment meant for neonatal care

Midwives recognised the need to be vigilant when caring for neonates immediately after birth. Some neonates required resuscitation, and where equipment was lacking or in poor repair, participants believed it contributed to some early neonatal deaths:

‘As a midwife, I am not only dealing with a pregnant mother. We are also dealing with a baby as well after the delivery.’ (P9, Female, Black person, Diploma in nursing & midwifery)

‘We will be resuscitating the baby in the ward … then you find that it is not well equipped.’ (P6, Female, Black person, Diploma in nursing & midwifery, Diploma in advanced midwifery)

‘You know what you have to do to save the baby’s life, but you don’t have the proper material. You end losing a baby because you just didn’t have enough to work with.’ P8, Female, Black person, Bachelor of nursing & midwifery)

An unfavourable hospital infrastructure, coupled with constraints in equipment, caused interruptions in resuscitation. Many of these newborns needed to be transported to the neonatal unit to ensure access to necessary equipment:

‘Our hospital isn’t well built to allow for continuity of care. We are a feeder to the neonatal units because they are supposed to admit all our sick neonates, but yoh! it is a real distance to walk there, they are on the other far right side of the hospital. We have no choice but have to rush there because, unlike us, at least they have some of the equipment to resuscitate the baby.’ (P4, Female, Black person, Diploma in nursing & midwifery, Diploma in advanced midwifery)

‘We need things like transport incubators that has all that we need to continue resuscitation while we are transporting the neonate because really our neonatal ICU [*Intensive care unit*] is far from [*the*] labour ward.’ (P5, Female, Black person, Diploma in nursing & midwifery)

Interruptions in the sequence of resuscitation during transport to the Neonatal intensive care unit (NICU) were believed to impact the chance of neonatal survival.

‘They say we are not supposed to accompany the patients in the ambulance, you find that you are referring a breech presentation, the paramedics are not skilled to perform that delivery, so we end up losing the neonate in case the woman delivers on the way to the hospital.’ (P4, Female, Black person, Diploma in nursing & midwifery, Diploma in advanced midwifery)

‘Our local hospital is plus or minus ten kilometers away from our MOU, but there is a delay with the ambulances. You have to wait for the ambulance to come and take the patient, which is also time consuming as well! By the time the patient is gone you are already drained and worried about the neonate!’ (P5, Female, Black person, Diploma in nursing & midwifery)

## Discussion

The study explored and described the avoidable causes of negative perinatal outcomes in the selected facilities in Bojanala District, North West Province, through the lens of midwives. Findings from this study suggest that midwives are aware of the avoidable causes contributing to escalating negative perinatal outcomes. By SANC registration for midwifery, participants should have the knowledge and skills to attend to complicated obstetric conditions. However, limited space and material resources restricted appropriate midwifery care. Participants detailed these constraints and identified them as avoidable causes of negative perinatal outcomes. The findings put the PPIP analysis of medical personnel–associated factors as avoidable factors under scrutiny. The medical personnel–associated avoidable factors focused more on the midwives’ knowledge and skills in managing the emergencies and less on the effect of resource constraints on management (Moodley et al. 2020). In instances where there is no evidence of medication administration or recording of specific vital signs due to equipment constraints, it is regarded as a medical personnel–related factor (DOH 2021). Such a determination should be reconsidered. Similarly, findings suggest the need for improved documentation on the part of the midwives (SANC 1990).

Results suggest that there were space constraints in admission, labour and high-care areas. Midwives stressed that these space constraints made it difficult to deal with the high volume of women seeking services. Space constraint within healthcare facilities, particularly in maternity departments, is a longstanding known problem (Medical Brief 2019). One media report detailed a hospital in Gauteng Province where 92 women occupied the maternity ward that had a capacity for 51 (Medical Brief 2019). Participants in this study complained that the majority of women accessing their facilities developed complications while waiting to be attended. They explained that some women had high-risk conditions and needed to occupy allocated rooms and beds for a significant amount of time while they were being monitored. This recognition by midwives is consistent with the research findings. Factors that contribute to increased wait times and a shortage of staff are major causes of poor perinatal outcomes (Kumari & Patyal 2018; Quaile 2018).

Limited access to life-saving drugs is a valid midwifery concern and is crucial in managing obstetric conditions. Notably, participants singled out magnesium sulphate as one of the most common drugs that is lacking on obstetrical units. The medication is essential in managing hypertensive disorders in pregnancy (Marshall & Raynor 2020). Lack of access to this drug restricts midwifery management of hypertensive disorders which affects 3% – 8% of all pregnancies (Marshall & Raynor 2020) and is one of the top five causes of maternal mortality (DOH 2016).

Midwives in this study stressed that constraints in medication supply limit a speedy and effective response to a variety of high-risk conditions such as preterm labour. Threatening conditions such as preterm labour that are not promptly treated pharmacologically contribute to poor perinatal outcomes. Failure to tocolyse a mother in preterm labour reduces foetal intrauterine time and an opportunity to promote foetal maturation in the uterus. Preterm labour accounts for a significant neonatal morbidity and mortality associated with complications of prematurity (Fozia et al. 2022). This finding regrettably contravenes the guidelines on immediate tocolysis of preterm labour. A pregnant woman between 26 and 33 weeks of gestation, experiencing preterm labour, is eligible for tocolysis to allow foetal lung maturity (Marshall & Raynor 2020).

Constraints in medical supplies necessary for midwifery care were significant participant concerns. Midwives explained that they often lacked basic resuscitation medical supplies such as oxygen masks. The oxygen mask serve has been an important medical supply where maternal and intrauterine foetal resuscitations are instituted (Kebeda & Kekulawa 2021). However, current researches have demonstrated that routine use of supplemental oxygen for foetal intrauterine resuscitation in women with normal oxygen saturation is no longer recommended, as there is no demonstrated benefit (American College of Obstetrics & Gynecology [ACOG] 2022). This finding suggests that many foetuses – irrespective of early diagnosis – may be subjected to prolonged intrauterine hypoxia that is inadequately treated or untreated altogether. As all foetuses run a risk of intrauterine hypoxia when uterine contractions are present (Marshall & Raynor 2020), the lack of key medical supplies is concerning.

The constraint in the supply of medical equipment necessary for maternal care and monitoring was a major concern for participants. They mentioned that the lack of medical equipment, such as equipment to measure blood pressure, makes it difficult to perform diagnostic procedures. The plight of participants was valid and substantiated claims by Jolobe (2022) that the correct diagnosis and classification of hypertensive-related disorders is dependent on the blood pressure reading. This was disappointing as it suggested that not all vital signs were monitored and limited the speedy and holistic response to women problems. This inappropriate response to a maternal condition is detrimental as hypertensive disorders in pregnancy cause multisystem involvement and damage when poorly managed (Marshall & Raynor 2020). While this finding endorsed midwives understanding of hypertensive disorders, it underscored the likelihood of delayed or undertreated management.

Participants also singled out the haemoglobin monitor as one of the constrained pieces of equipment in their respective units. The inability to evaluate for anaemia and its related complications, which include foetal distress and maternal haemorrhage (Marshall & Raynor 2020), is a shortcoming of the system. The inability to evaluate for anaemia may contribute to poor perinatal outcomes as it is known that it is a significant risk factor for preterm labour (Fozia et al. 2022). The presence of anaemia predisposes the foetus to intrauterine growth restriction and foetal distress secondary to limited supply of blood rich in oxygen and nutrients (Marshall & Raynor 2020). Consideration of the potential for anaemia by participants was important and consistent with maternity care guidelines (DOH 2016). The constraints in resources necessary to monitor foetal well-being were a significant participant concern. Foetal monitoring is a diagnostic aid during episodes of intrauterine hypoxia and signals the need for immediate intrauterine resuscitation and/or plans for birth (Aziz et al. 2021). The plight of midwives, where equipment for foetal surveillance was lacking, was clearly articulated and mandates the need for systems to prioritise equipment (e.g. electronic foetal monitors, dopplers, fetoscopes or Pinard horns). The potential for foetal compromise during labour is a serious consideration as limited blood flow may result in foetal distress impacting the neonate’s survival rate (Aziz et al. 2021).

Another major concern was the marked constraint in medical equipment necessary for quality neonatal care. Participants were appreciative of the need to resuscitate high-risk neonates within the first minute of life, as well as immediate access to the neonatal intensive care unit to prevent early neonatal deaths (Aziz et al. 2021). A delay in resuscitation of neonates has detrimental effects and accounts for a significant percentage of neonatal deaths (Vallely et al. 2021). Access to adequate equipment may avert such negative perinatal outcomes.

## Strengths and limitations

The study highlights the importance of utilisation of the PPIP tool, to reveal the avoidable causes of negative perinatal outcomes, and maternal and neonatal mortalities by the midwives. Midwives are primary caregivers for pregnant women, and their use of the PPIP tool provides an opportunity for reflection on their own professional midwifery practice and improvement in the quality of care rendered. While the study examined the experiences of midwives from two select sites, other types of maternity care providers were not included, and all participants were black and female which limited generalisability. In addition, none of the participants held advanced midwifery degrees and had substantial clinical experience. Additional research is yet needed to ascertain how other maternity care providers (e.g. physicians) describe contributing factors to negative perinatal outcomes.

## Conclusion

This research examined the description of midwives regarding avoidable causes contributing to negative perinatal outcomes as viewed within the context of the PPIP survey. A qualitative, exploratory and descriptive study design was used to detail participants’ experiences, which could be used to elicit the experience of midwives in identifying avoidable causes of negative perinatal outcomes.

Space and resource constraints were clearly identified as avoidable causes of negative perinatal outcomes, as were maternal and neonatal mortalities. The challenges experienced and referred to as avoidable causes of negative outcomes can serve as a guide in the managerial decision-making within the midwifery and obstetric context. The outcome of this study can provide a guide in the planning, requisition and allocation of resources necessary for maternity care. The midwives in this study were knowledgeable about PPIP, recognising the avoidable causes of negative perinatal outcomes.

Findings underscore the need to address administrative-related avoidable causes of negative perinatal outcomes. Management would be well served to consider working collaboratively with midwives to ensure adequate supplies and services necessary for safe and effective midwifery care.

## References

[CIT0001] American College of Obstetrics & Gynecology (ACOG), 2022, *Oxygen supplementation in the setting of category II or III foetal heart tracings*, Practice Advisory, viewed 06 February 2022, from https://www.acog.org/clinical/clinical-guidance/practice-advisory/articles/2022/01/oxygen-supplementation-in-the-setting-of-category-ii-or-iii-foetal-heart-tracings.

[CIT0002] Aziz, K., Lee, H.C., Escobedo, M.B., Hoover, A.V., Kamath-Rayne, B.D., Kapadia, V.S. et al., 2021, ‘Part 5: Neonatal resuscitation 2020 American Heart Association guidelines for cardiopulmonary resuscitation and emergency cardiovascular care’, *Pediatrics* 147(Suppl 1), e2020038505E. 10.1542/peds.2020-038505E 33087555

[CIT0003] Bera, S., Deb, D., Ballov, R., Chakraborty, S., Ballov, R. & Chakraborty, S., 2022, ‘Efficacy of admission cardiotocography on neonatal outcome in term pregnancy admitted in labor in a rural-based tertiary care center: A prospective study’, *Asian Journal of Medical Sciences* 13(6), 139–144. 10.3126/ajms.v13i6.42856

[CIT0004] Creswell, J.W. & Poth, C., 2018, *Qualitative inquiry and research design, choosing among five approaches*, 4th edition. New Dehli: Sage.

[CIT0005] Department of Health South Africa, 2016, *Guidelines for maternity care in South Africa*, Department of Health, Pretoria.

[CIT0006] Department of Health South Africa, 2021, *South African maternal, perinatal and Neonatal health policy*, viewed 15 June 2022, from https://www.knowledgehub.org.za/system/files/elibdownloads/2021-06/SA%20MPNH%20Policy%2023-6-2021%20signed%20Web%20View%20v2.pdf.

[CIT0007] Department of Health South Africa, n.d., *The perinatal problem identification program v2.0*, Department of Health, Pretoria, viewed 15 June 2022, from http://www.kznhealth.gov.za/paed/PPIP/ppip2.pdf.

[CIT0008] Du Plessis, D., Grobler, A. & Seekoe, E., 2020, *The experiences of private midwive’s when implicated in a medical negligence claims*, viewed 07 September 2021, from https://www.dianaduplessis.co.za/medical-negligence/.

[CIT0009] Fozia, A., Bibi, H., Nasir, S., Khan, M., Asmat, L. & Anwar, Z., 2022, ‘Frequency of anemia in patients with preterm labour’, *Professional Medical Journal* 29(5), 658–662. 10.29309/TPMJ/2022.29.05.6830

[CIT0010] Heitkamp, A., Aronson, S.L., van den Akker, T., Vollmer, L., Gebhardt, S., van Roosmalen, J. et al., 2020, ‘Major obstetric haemorrhage in Metro East, Cape Town, South Africa: A population-based cohort study using the maternal near-miss approach’, *BMC pregnancy and childbirth* 20(1), 14. 10.1186/s12884-019-2668-x. 31906889 PMC6945549

[CIT0011] Jolobe, O., 2022, ‘The differential diagnosis of pre-eclampsia should include the association of severe hypertension and aortic dissection’, *Clinical Medicine (London, England)* 22(1), 92. 10.7861/clinmed.Let.22.1.1 PMC8813023 35078803

[CIT0012] Kebede, E. & Kekulawala, M., 2021, ‘Risk factors for stillbirth and early neonatal death: a case-control study in tertiary hospitals in Addis Ababa, Ethiopia’, *BMC pregnancy and childbirth* 21(1), 641. 10.1186/s12884-021-04025-8. 34548064 PMC8456546

[CIT0013] Kumari, N. & Patyal, S., 2018, ‘Waiting time: Perception of demographic groups’, *International Journal on Customer Relations* 6(1), 6–10.

[CIT0014] Lavin, T., Allanson, E.R., Nedkoff, L., Preen, D.B. & Pattinson, R.C., 2018, ‘Applying the international classification of diseases to perinatal mortality data, South Africa’, *Bulletin of the World Health Organization* 96(12), 806–816. 10.2471/BLT.17.206631 30505028 PMC6249699

[CIT0015] Lincoln, Y.S. & Guba, E.G., 1986, ‘But is it rigorous? Trustworthiness and authenticity in naturalistic evaluation’, *New Directions for Program Evaluation* 30(Summer) 73–84. 10.1002/ev.1427.

[CIT0016] Marombwa, N.R., Sawe, H.R., George, U., Kilindimo, S.S., Lucumay, N.J., Mjema, K.M. et al., 2019, ‘Performance characteristics of a local triage tool and internationally validated tools among under–fives presenting to an urban emergency department in Tanzania’, *BMC Paediatrics* 19(1), 44. 10.1186/s12887-019-1417-7 PMC6357459 30709389

[CIT0017] Marshall, J. & Raynor, M., 2020, *Myles textbook for midwives*, 17th edn., Elsevier, Edinburgh.

[CIT0018] Medical Brief, 2019, *Hospital overcrowding: 192 Instead of 51 in maternity ward*, viewed 10 December 2022, from https://search.ebscohost.com/login.aspx?direct=true&AuthType=sso&db=awn&AN=1398722&site=ehost-live&scope=site.

[CIT0019] Moodley, J., Fawcus, S. & Pattinson, R., 2020, ‘21 Years of confidential enquiries into maternal deaths in South Africa: Reflections on maternal death assessments’, *Obstetrics & Gynaecology Forum* 30(4), 4–7, viewed 15 June 2022, from https://search.ebscohost.com/login.aspx?direct=true&AuthType=sso&db=asn&AN=147820721&site=ehost-live&scope=site.

[CIT0020] Polit, D.F. & Beck, C.T., 2017, *Nursing research*, Generating and assessing evidence for nursing practice, 10th edn., Wolters Kluwer, Philadelphia, PA.

[CIT0021] Polit, D. & Beck, C., 2019, *Nursing research*, 11th edn., Wolters Kluwer Health, Philadelphia, PA.

[CIT0022] Quaile, H., 2018, ‘Implementing an obstetric-specific triage acuity tool to increase nurses knowledge and improve timelines of care’, *Clinical Innovations: Childbearing* 22(4), 293–301. 10.1016/j.nwh.2018.05.002 30077235

[CIT0023] Sawicka-Gutaj, N., Gruszczyński, D., Guzik, P., Mostowska, A. & Walkowiak, J., 2022, ‘Publication ethics of human studies in the light of the Declaration of Helsinki – A mini-review’, *Journal of Medical Science* 91(2), 81–85. 10.20883/medical.e700

[CIT0024] South African Nursing Council (SANC), 1990, *Regulations relating to the conditions under which registered midwives carry on their profession*, Regulation No. R.2488 of 1990 as amended, SANC, Pretoria, viewed 13 August 2022, from https://www.sanc.co.za/r-2488/

[CIT0025] Statistics South Africa, 2022, *Maternal mortality rate on the decline in SA*, Department of Health, Pretoria, viewed 15 June 2022, from https://www.statssa.gov.za/?p=15321#:~:text=Nationally%2C%20the%20ratio%20decreased%20from,experiencing%20a%20decrease%20in%20MMFR.

[CIT0026] Thapa, J. & Sah, R., 2017, ‘Admission cardiotocography in high risk pregnancies’, *Nepal Journal of Obstetrics & Gynaecology* 12(1), 50–54. 10.3126/njog.v12i1.18982

[CIT0027] Vallely, L.M., Smith, R., Laman, M., Riddell, M.A., Mengi, A., Au, L. et al., 2021, ‘Early neonatal death review from two provinces in Papua New Guinea: A retrospective analysis’, *Journal of Pediatrics and Child Health* 57(6), 841–846. 10.1111/jpc.15333 33450113

[CIT0028] WHO, 2022, *WHO recommendations: Maternal and perinatal death surveillance and response: Materials to support implementation*, viewed 12 February 2022, from https://apps.who.int/iris/rest/bitstreams/1390203/retrieve.

